# Impact of composition and surfactant-templating on mesoporous bioactive glasses structural evolution, bioactivity, and drug delivery property

**DOI:** 10.1177/08853282241312040

**Published:** 2025-01-08

**Authors:** Dana Almasri, Yaser Dahman

**Affiliations:** 1 Biomedical Engineering Graduate Program, 7984 Toronto Metropolitan University, Toronto, ON, Canada; 2 Department of Chemical Engineering, Toronto Metropolitan University, Toronto, ON, Canada

**Keywords:** Mesoporous, bioactive glass, drug loading, drug release, kinetic modelling, surfactants, chemical synthesis, sol-gel, and bioactivity

## Abstract

This study explores mesoporous bioactive glasses (MBGs) that show promise as advanced therapeutic delivery platforms owing to their tailorable porous properties enabling enhanced drug loading capacity and biomimetic chemistry for localized, sustained release. This work systematically investigates the complex relationship between MBG composition and surfactant templating on structural evolution, *in vitro* bioactive response, resultant drug loading efficiency and release. A total of 12 samples of sol-gel-derived MBG were synthesized using cationic and non-ionic structure-directing agents (cetyltrimethylammonium bromide, Pluronic F127 and P123) while modulating the SiO_2_/CaO content, generating MBG with surface areas of 60–695 m^2^/g. Electron microscopy and nitrogen desorption studies verified the successful synthesis of the 12 ordered MBG formulations. Assessment of hydroxyapatite conversion kinetics via FTIR spectroscopy and SEM demonstrated accelerated deposition for 70–80% SiO_2_ formulations, independent of the surfactant used. However, the templating agent had an impact on drug loading as observed in this study where MBG synthesized by the templating agent Pluronic P123 had higher drug loading compared to the other surfactants. To determine the drug release mechanisms, the in vitro kinetic profiles were fitted to various mathematical models including ze-ro. Most compositions exhibited release properties closest to zero-order, indicating a concentration-independent drug elution rate. These results in this study explain the relationship between tailored hierarchical architecture and intrinsic ion release rates to enable advanced functionality.

## Introduction

Bioactive glass is a synthetic ceramic biomaterial first developed in the 1960s by Hench^1^ and has recently gained significant interest owing to its flexible synthesis methods and diverse range of applications.^2^ One key area being explored is the use of bioactive glasses for the local delivery of antibiotics, which involves the continuous release of antibiotics directly at the infection sites. In order to safely achieve treatment goals of eliminating bacteria, a reliable drug carrier system is needed to allow for effective treatment without toxicity.^3^ Bioactive glass has emerged as a promising platform owing to its biocompatibility, bioactivity, and ability to form a bone-like apatite layer when it is exposed to bodily fluids.^4,5^ The biodegradability of bioactive glass facilitates dissolution within the body, allowing the deposition of hydroxyapatite (HCA) that integrates with bone to stimulate growth.^4^ Mesoporous bioactive glass (MBG), a subclass with organized porosity, possesses improved characteristics. They are typically synthesized via a versatile sol-gel technique using surfactants as surface-directing agents to create well-defined porous structures.^5^ This has expanded the applications of bioactive glass in fields such as controlled drug delivery, implant coatings, and bone scaffolds, showing remarkable versatility.^6^

MBG is typically synthesized through the sol-gel process, in which a surfactant is dissolved in either water or ethanol, followed by the addition of metal oxide precursors in acidic or basic solutions. The resulting solutions are poured into Petri dishes, where gel formation is initiated.^7^ The gel can be allowed to dry naturally at room temperature or in an oven, after which it is calcined at high temperatures to form the final powder. It is important to note that calcination of MBG is necessary to remove any surfactants or contaminants from the material and improve its stability.^8,9^

The pores within MBG are created using surface-directing agents, typically in the form of chemical surfactants. These surfactants are amphiphilic compounds that can form micelles at high concentrations in solution.^10,11^ When synthesizing MBG with a surfactant, the surfactant functions as a surface-directing agent, creating pores in the MBG by assembling into micelles with a hydrophilic outer region and hydrophobic inner region. The size of the pores and the property of the resulting glass depends on the specific surfactant used, as surfactants self-assemble in micelles at their critical micelle concentration (CMC).^11^ This study aimed to investigate the impact of surfactant selection on MBG synthesis as well as the properties of the resultant MBG. Three common surfactants for templating MBG were selected: (1) Pluronic P123, a non-ionic poly(ethylene oxide)-poly(propylene oxide)-poly(ethylene oxide) triblock copolymer with the composition (PEO)_20_(PPO)_70_(PEO)_20_^12^; (2) Pluronic F127, also a non-ionic Pluronic surfactant but with longer PEO chains ((PEO)_106_(PPO)_70_(PEO)_106_)^11^; and (3) cetyltrimethylammonium bromide (CTAB), a positively charged cationic surfactant.^13^ Pluronic P123 has been extensively used as a structure-directing agent in MBG fabrication. Meanwhile, counterparts F127 and CTAB possess unique advantages for tailored mesostructured syntheses, such as surface charge modulation (cationic CTAB) and enhanced stabilization against aggregation effects (PEO-rich Pluronic F127).^11^ The size and texture of the pore created by the template directly impacts the properties of the MBG produced. While some reviews show an overview of how various templates impact MBG properties, there is a lack of articles that directly compares the properties of MBG synthesized using different surfactants.^8^ The choice of surfactants can result in pores with distinct geometries including hexagonal, lamellar or radial which will have an impact on drug loading and release. Comparing the performances of these three surfactants will help determine the differences and inform the selection of optimal surfactant templates in future studies.^11–13^

Another important factor in determining the properties of MBG is its composition, which consists of network formers and modifiers. Network formers, such as silicate and phosphate, are integral components of the chemical structure of bioactive glass. During the synthesis of MBG, alkoxysilanes undergo hydrolysis, leading to the formation of silanol groups that subsequently react with each other or with alkoxysilanes to form siloxanes during the condensation step of the reaction.^11,14^ The siloxane bond (Si-O-Si) is a covalent bond that creates bridging oxygen atoms. The siloxane network can be altered by the addition of network modifiers, such as calcium, which introduces non-bridging oxygens, resulting in a “weaker” network with a faster degradation rate. Phosphate, another component of the glass network, can function as a network former similar to silicate and influences the reactivity of the glass.^15^ The objective of this study was to examine the effects of varying the surfactant template and bioactive glass composition on the subsequent loading capacity and release of antibiotics as model drugs. This study examined the coupled effects of changing the SiO_2_ and CaO composition of MBG on tailoring critical drug delivery attributes of the mesoporosity and biodegradation profile that govern biosorption and release of therapeutics.

Biocompatibility of bioactive glasses has been supported by studies such as Chen et al. which tested cell viability *in vitro* where they assayed 58S BG by cultivating MC3T3 osteoblasts cells with bioactive glass and observed viability of cells. The study found that cells grow well after culturing with BG tablets.^15^ Another study by Anand et al. where they tested biocompatibility of MBG *in vivo* and found that all samples they tested led to formation of new bones and specifically MBG-CTAB which led to new bone formation at 68.2% after 90 days.^13^ Another comprehensive review conducted by Saletes et al. examined the biocompatibility of MBGs based on *in vitro* studies. The review systematically analyzed the various approaches taken to assess the biocompatibility of MBGs. According to the findings of this review, the majority of the studies evaluated reported that MBGs demonstrate favorable biocompatibility, cytocompatibility, osteoinductive properties, and lack of cytotoxicity.^16^ The thorough analysis presented in the review provides strong evidence supporting the biocompatible nature of MBGs when tested *in vitro*^17^. Accordingly, findings from this study highlight the potential of MBG as a promising material for biomedical applications, particularly in the context of bone tissue engineering and local drug delivery.

This study focuses on the critical interplay between changes in chemical composition and the use of surfactants in influencing the properties MBGs and their drug release kinetics. Despite the growing interest in MBGs as drug delivery systems, the literature lacks comprehensive investigations into how variations in surfactant types and compositions affect their drug-loading and release capabilities. Given that MBGs—and bioactive glasses in general—are highly sensitive to synthesis conditions, this study seeks to bridge the identified research gaps by systematically evaluating the effects of these variables. The aim is to identify optimal synthesis approaches that enhance MBG properties for effective drug delivery applications.

## Materials and Methods

Tetraethyl orthosilicate (TEOS) (99%) was used as a silica source. Triethyl phosphate (TEP) (99%) and calcium nitrate tetrahydrate (CaN) (99%) were used as the sources of phosphate and calcium, respectively. The structure-directing agents used were the non-ionic copolymer surfactant Pluronic F127 (powder), Pluronic P123 (viscous powder), and hexadecyltrimethylammonium bromide (≥99%). Ethanol (reagent alcohol >99%) water (Millipore deionized), hydrochloric acid, HCl (ACS reagent >37%), and nitric acid (10N) were used as the solvents. Phosphate-buffered saline (PBS) tablets dissolved in deionized water were used for the drug release studies. All materials were purchased from Sigma-Aldrich and used without further modifications. Vancomycin hydrochloride (sterile for injection, USP) used in the experiment was gifted by Mount Sinai Hospital.

### Experimental procedure for synthesising MBG

Twelve MBG samples were synthesized with different compositions and surfactants. Three surfactants were examined: Pluronic F127(MBG-F), Pluronic P123 (MBG-P), and CTAB (MBG-C). For each of these surfactants, four compositions were synthesized by varying the SiO_2_ and CaO contents; the samples with different compositions are listed in Table 1. The compositions were selected to examine the impact of introducing non-bridging oxygens into the MBG network and to determine if compositions play a significant role in dissolution of bioactive glasses.

**Table 1.** Nominal composition of MBG samples.

Sample name	mol (%)			
	Surfactant	SiO_2_	CaO	P_2_O_5_
MBG-P85	Pluronic P123	85	10	5
MBG-P80	Pluronic P123	80	15	5
MBG-P70	Pluronic P123	70	25	5
MBG-P60	Pluronic P123	60	35	5
MBG-C85	CTAB	85	10	5
MBG-C80	CTAB	80	15	5
MBG-C70	CTAB	70	25	5
MBG-C60	CTAB	60	35	5
MBG-F85	Pluronic F127	85	10	5
MBG-F80	Pluronic F127	80	15	5
MBG-F70	Pluronic F127	70	25	5
MBG-F60	Pluronic F127	60	35	5

The following procedure describes the synthesis procedure of 80SiO_2_-15CaO-P_2_O_5_. Typical synthesis procedure for MBG-P began with dissolving 4.0 g of P123 in a solvent mixture consisting of 60 g ethanol and 1 g of 0.5 M HCl under vigorous stirring until fully dissolved.^10,18^ The metal oxide precursors were then added sequentially under continuous stirring: first, 6.8 g of TEOS as the silica source, followed by 1.4 g CaN as a source of calcium after 1 hour. One hour later, 0.73 g of TEP was added as the source of phosphate.^18^ The solution was stirred overnight to allow complete hydrolysis and condensation of the silicate network. Finally, the mixture underwent evaporation-induced self-assembly (EISA) by pouring it into dishes at room temperature.^19^ Finally, calcination at 600°C (1°C/min heating rate) was performed in a Lindberg Furnace (Thermo Scientific, USA) to remove the surfactant templates and produce MBG powder.^18^ The as-synthesized MBG was crushed, sieved to 63 μm particles, and processed with other surfactants and compositions using the same methodology. For MBG-F, the same procedure was followed, using 4.0 g of Pluronic F127 surfactant, as described in Shih et al.,^11^ For MBG-C, the procedure outlined in Anand et al.^13^ was used, where approximately 106 mL of ammonia and 1L of DW were mixed followed by the addition of 2.64 g of CTAB, which was stirred until fully dissolved. Subsequently, 8.53 g of TEOS was added, followed by 2.55 g of CaN and finally 0.66 g of TEP, with an hour internal between each addition

### Characterization

#### X-ray diffraction

Powdered samples of MBG were used to collect XRD patterns on a MiniFlex 600 diffractometer (Rigaku Corporation, Japan) under Cu Kα radiation (40 kV, 15 mA) to determine crystallinity. The instrument was set to take measurements between 5° and 90° at an increment of 2°. The data for as-synthesized MBG is displayed from 5° to 90° while the bioactivity data is displayed from 20° to 70°.

#### Fourier transform infrared spectroscopy (FTIR)

FTIR on a Cary 630 spectrometer (Agilent, USA) was used to assess molecular vibrations using the KBr pellet method from 4500 to 550 cm^−1^, before and after immersion in SBF. The MBG samples were immersed in SBF to evaluate the growth of HCA nanocrystals on the material surface as a measure of bioactivity. Each glass sample was filtered and dried at predetermined times and then analyzed using FTIR following protocols established in previous studies.^6–9^

#### Inductively coupled plasma optical emission spectrometry (ICP-OES)

Ion concentrations released into the SBF were quantified by ICP-OES on an Agilent 5900 system (Agilent, USA) at various time points to study degradation. ICP-OES a is used to measure the degradation and bioactivity of the MBG synthesized in this study. It consists of analyzing the concentrations of calcium, phosphorus, and silicon following the placement of MBG in SBF for different periods. In this study, 2.5 mL of the sample was removed and added to nitric acid to eliminate potential interference and contamination. The samples were collected at 6 h and on days 1, 2, 10, and 20 to show the overall ion release trends.

#### BET surface area

Nitrogen adsorption analysis was performed to measure the surface area, pore volume, and pore size using a TriStar 3000 porosimeter (Micromeritics, USA) and the BET method. A sample of 0.3 g was used for analysis, with each sample degassed at 200°C for 12 h to clear up the pores and allow for accurate measurements.

#### Scanning electron microscopy (SEM)

SEM JSM-6380LV (JEOL, Japan) was used to characterize the morphology and structure of the samples after SBF exposure. The MBG samples were immersed in SBF at a ratio of 1.5 mg/mL and left in a water bath in a shaker at 37°C for 6 h, 1, and 7 days. The samples were then filtered and air-dried to investigate the development of HCA on the surface. Prior to analysis in the SEM the samples were coated with one layer of gold.

### In vitro bioactivity

A simulated body fluid (SBF) solution was prepared by dissolving reagent-grade NaCl, KCl, NaHCO_3_, MgCl_2_·6H_2_O, CaCl_2_, and KH_2_PO4·3H_2_O in distilled water. The solution was buffered at pH 7.4, using Tris (hydroxymethyl) aminomethane (TRIS) and 6N HCl at 37°C, according to the Kokubo protocol.^20^ The biomimetic SBF solution exhibited an ionic composition comparable to that of human blood plasma. It enables the *in vitro* evaluation of bioactive glass dissolution and apatite formation under physiologically relevant conditions.^21,22^ To assess dissolution, 15 mg of MBG powder was suspended in 10 mL of SBF at a ratio of 1.5 mg/mL and kept at 37°C with constant stirring in a water bath shaker for 30 days. The tubes were then centrifuged and filtered to recover MBG, which was fully dried prior to analysis by FTIR spectroscopy. The supernatant was analyzed using ICP-OES, as described above.

### Drug loading and in vitro release profile

Vancomycin and ciprofloxacin were used separately as model drugs and loaded onto mesoporous carriers using a simple impregnation method.^21,23^ Briefly, 200 mg of the MBG samples were added to 20 mL of antibiotic solutions prepared at 5 mg/mL in deionized water. The mixtures were stirred continuously for 24 h to facilitate drug adsorption onto mesopores with high surface area bioglass particles. The MBGs were then filtered, air-dried and the residual concentrations of vancomycin and ciprofloxacin in the filtrate were measured using UV-visible spectroscopy at wavelengths of 280 nm and 277 nm, respectively, to determine depletion.^24–26^ Standard calibration curves were constructed for both model drugs.

A standard calibration curve for vancomycin was used to determine the concentration of the remaining vancomycin after loading, and the linear range of 20–600 μg/mL was used as it had a correlation coefficient of 0.99. The standard curve for ciprofloxacin was over a range of 1–30 μg/mL with a correlation coefficient of 0.99. The percentage of antibiotics loaded into the MBGs was calculated by comparing the initial and final concentrations using the following equation.

$$Drug\;Loading\;(\%)=\frac{\left(C_{i}-C_{f}\right)}{C_{i}}\times 100\%\;$$

Where Ci and Cf are the initial and final concentrations, respectively. The successful incorporation of antibiotics into the bioglass samples was validated using FTIR. For consistency, an 80% SiO_2_ composition was used to compare the effects of the three different surfactant template loading abilities (see Table 1). This comparison can help evaluate how the tailorable mesopore texture and chemistry of these bioactive glass carriers govern the critical performance metric of drug loading capacity.

Drug release experiments were performed by immersing 50 mg of drug-loaded bioactive glass samples in 10 mL of PBS in plastic tubes. The tubes were then placed in an incubator at 37°C with continuous stirring for 24 h to mimic the physiological conditions. At 10-min intervals, 1 mL of the release medium was collected for analysis using UV-visible spectroscopy to quantify the amount of drug released. To maintain the drug concentration gradient driving the release, the removed volume was replenished with an equal volume of fresh PBS solution.

To assess the release mechanisms and kinetics of vancomycin from the MBG compositions *in vitro*, the cumulative drug release data were fitted to various mathematical models.^27,28^ By comparing the goodness of fit to different models, the predominant release kinetics can be determined. The models applied in this study include:

#### Zero-order model

Assumes uniform drug release rate independent of concentration. It is calculated using the following equation:

$$C_{t}=C_{0}+kt$$

where C_t_ is the amount of drug dissolved at time t, C_0_ is the initial amount of drug in solution, and k describes the zero-order rate constant.

#### First-order model

Release rate dependent on concentration in a linear manner. It is calculated using this equation:

$$\frac{dC}{dt}=-kC$$

which can also be expressed as:

$$\log\;C=\log\;C_{0}-\frac{Kt}{2.303}$$

where C_0_ is the initial drug concentration and k describes the first-order rate constant.

#### Higuchi model

Describes drug release from matrix systems based on Fickian diffusion. It is calculated using the following equation:

$$f_{t}=Q=A\sqrt{D\left(2C-C_{s}\right)C_{s}t}$$

where Q refers to the amount of drug released in time t. A is the unit area, C is the drug initial concentration, and the drug solubility is represented by C_s_. D refers to the diffusivity of the drug.^29^

The Higuchi Model can be simplified to describe drug release from matric and polymeric systems as follows:

$$\frac{M_{t}}{M_{\infty }}=k\surd t$$

where M_t_/M_¥_ is the cumulative amount of drug released at time t, and k is the Higuchi constant.

#### Korsmeyer-Peppas

Empirical model combining diffusion and erosion effects. It is calculated using the following equation:

$$\frac{M_{t}}{M_{\infty }}=k^{\prime }t^{n}$$

where M_t_/M_¥_ is the cumulative amount of drug released at time t, $k^{\prime }$ is the kinetic constant, and *n* is usually used to describe a specific diffusion mechanism. The exponent *n* is used to describe the diffusion mechanism of the system; where it represents Fickian diffusion if *n* = 0.5, non-Fickian transport if 0.45 < *n* = 0.89. If *n* = 0.89 then it is zero order release (case II) transport. If *n* is higher than 0.89 then diffusion mechanism is Super case II transport.^29^

Fitting the vancomycin release profiles to these models enables identification of the predominant kinetics whether they be concentration-dependent, diffusion-based, or relaxational. Furthermore, the impact of composition and surfactant templating on the release mechanism can be explained by comparing model fitting between MBG formulations.

### Antimicrobial susceptibility test

The antibacterial activity of MBG samples was evaluated against *Staphylococcus aureus* (ATCC 25923) and *Escherichia coli* (ATCC 25922) using the disc diffusion method as described in the British Society for Antimicrobial Chemotherapy (BSAC) Disc Diffusion Method for Antimicrobial Susceptibility Testing, Version 4 (2005).^30^ Hinton Mueller agar plates were used as the testing medium. Bacterial strains (*S. aureus* and *E. coli*) were prepared by suspending them in saline solution to achieve a suitable inoculum density. A volume of 50 µl of bacterial inoculum was evenly spread across the surface of the agar plates and allowed to dry for a few minutes to ensure proper adherence. Following the preparation of the plates, 50 µg of MBG sample was placed into wells on the agar plates. The plates were incubated at 37°C for 24 hours to allow for bacterial growth and interaction with the MBG samples. After the incubation period, the zone of inhibition (ZOI) surrounding each MBG sample was measured to determine the antibacterial efficacy.

## Results and Discussions

### Characterization of the MBGs

**Figure 1.**
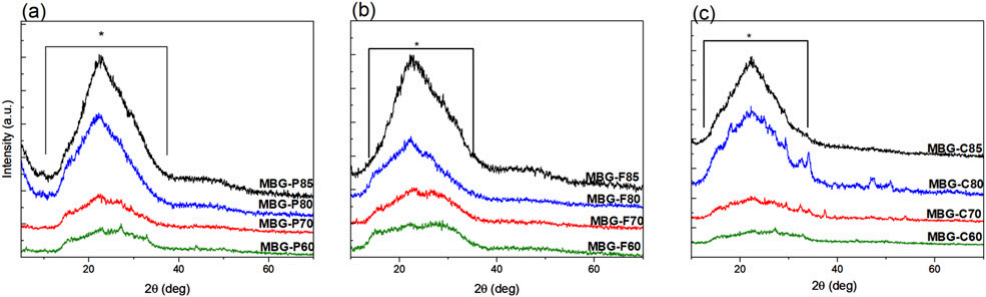
X-ray patterns of (a) MBG-F including all the different compositions as indicated in the figure, (b) MBG-C and all its different compositions, and (c) MBG-P also including all its compositions. The Figure shows that sol-gel synthesized bioactive glass samples have an amorphous structure indicated by the broad dispersive peaks. Each figure shows the highlighted region where the peaks start and end around 10° to 30° on average for MBG-F85, MBG-C85 and MBG-P85.

Figure 1 shows the X-ray patterns of different MBG compositions which verified the nanocrystalline amorphous nature of the MBGs. The figure shows broad dispersive peaks which are characteristic of silicate glasses.^17,29^ The broad peaks are observed at 2**θ** ∼10–38° and are highlighted on the figure to show the extent of the dispersive peaks.^31^ Figure 1(a) shows the diffraction patterns for MBG-P compositions, showing a broad amorphous dispersive peak around 10–38° this dispersive peak is most intense for the MBG-F85 followed by MBG-F80, MBG-F70 then MBGF60. It is imperative to note that as the silica content decreases the dispersive peak decreases in intensity. Figure 1(b) shows a similar trend of the dispersive peaks decreasing in intensity as the composition decrease in silica content for MBG-F. Figure 1(c) shows the diffraction patterns for MBG-P which similar to the others shows a higher intensity broad peak around 10–38° that is less intense with decreasing silica content but with microcrystalline phases observed in the MBG-C80 but not the other compositions.

Since it was observed that similar broad spectral signatures are observed across the same compositions synthesized using different templates, which indicates that the surfactant has less impact on the amorphous structure of the MBG than the compositional variations. Further, when comparing across compositions, those with higher silica content (80–85 mol% SiO_2_) exhibited more defined scattering profiles with distinct broad peaks, likely from increased molecular ordering of the predominant network former SiO_2_ phase.^17^ Conversely, glasses with a lower 60–70 mol% SiO_2_ showed flatter profiles lacking distinct features, suggesting more disordered arrangements as CaO replaces SiO_2_.^31^ With a higher CaO content in the composition, the bridging oxygens are replaced with non-bridging oxygens, which could explain the disordered arrangement of the glass particles.^31–34^ Nevertheless, most formulations match the amorphic signatures expected, without sharp crystalline diffraction peaks confirming successful sol-gel synthesis of largely homogenous, glass particles across the MBG compositions.

### In vitro bioactivity

**Figure 2.**
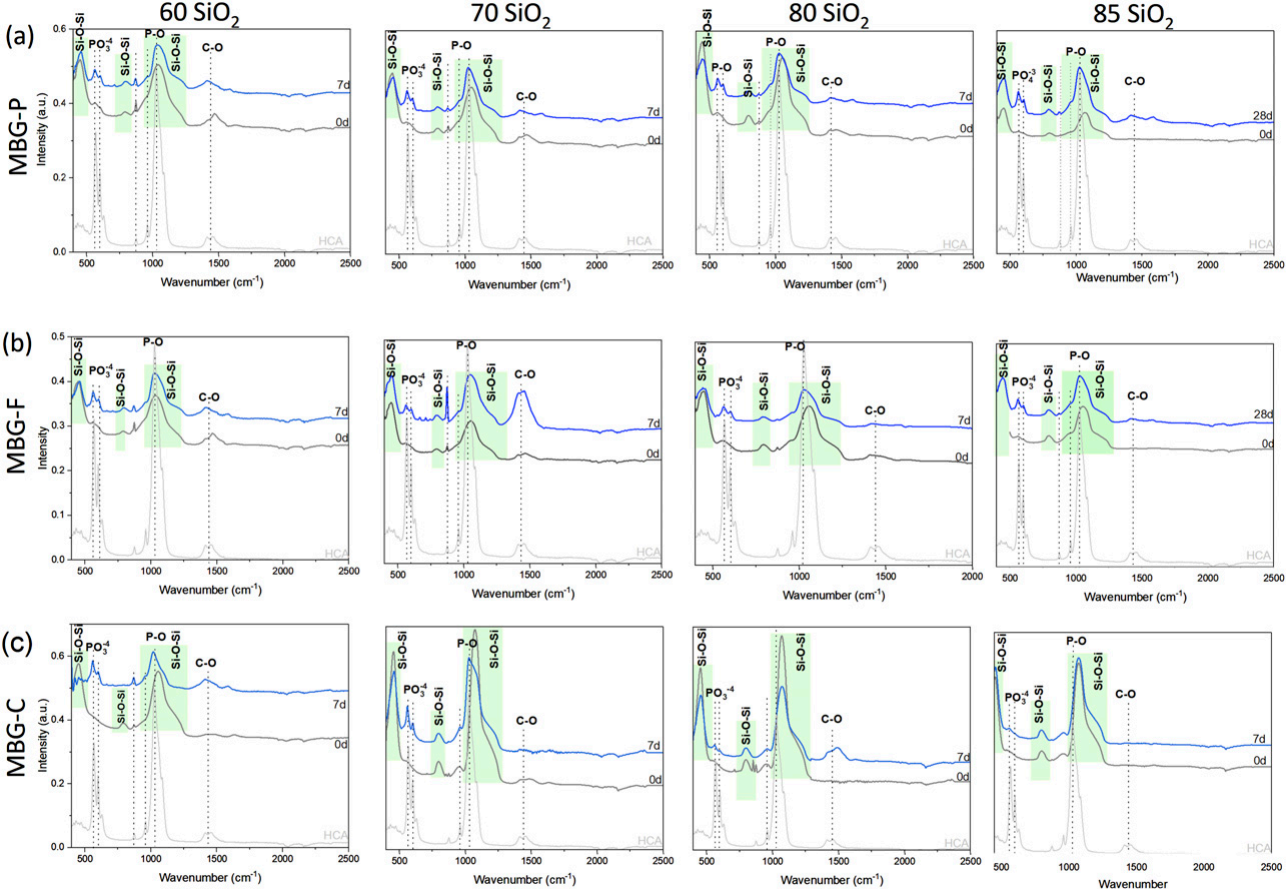
FTIR spectra of MBG-P, MBG-F and MBG-C before and after immersion in SBF for 7 days. The figure shows the development of HCA on the surface of MBG with time. MBG-P shows the most discernable peaks while MBG-C shows the lowest intensity peaks observed.

FTIR analysis illustrated in Figure 2 demonstrates the evolution of HCA on the surface of MBG after immersion in SBF for 7 days. Figure 2(a) shows the spectra for MBG-P with different compositions where each graph shows the FTIR spectra at day 0 and compares it to the spectra at day 7 to show the development of HCA on the surface of MBG. Regions highlighted in green show Si-O-Si bonds while dotted lines highlight the peaks observed after immersion in SBF. Figure 2(b) and (c) show the FTIR spectra of MBG-F and MBG-C compositions.

The highlighted bands in the FTIR spectra correspond to characteristic peaks of HCA at 570.2827, 600.1014, 1028.745, 1412.661 and 1453.6618 cm^−1^, representing the functional groups of HCA.^35,36^ By comparing the FTIR spectra of HCA with those of MBG, specific functional groups associated with HCA are identified in the MBG samples, indicating the development of HCA on the MBG surfaces. The highlighted peaks in the FTIR spectra correspond to specific functional groups characteristic of HCA. Phosphate groups are indicated by peaks appearing at 558–607, 930–960, and 1028–1039 cm^−1^ which are observed after 7 days of immersion in SBF. Specifically, the band appearing at around 558–607 cm^−1^ corresponds to the bending mode of crystalline phosphate P-O and the peak at 1028–1039 cm^−1^ indicate stretching mode of P-O. Additionally, the peaks highlighted at 1412–1472 cm^−1^ represent C-O stretching vibrations.^34^

These distinctive peaks confirm the presence of HCA on the MBG surfaces after immersion in SBF. Glass dissolution is evidenced by the shift in peaks observed around 1050–1070 cm^−1^ for most formulations after immersion in SBF for several days. For example, in MBG-P70, the Si-O-Si peak shifted from 1047.3819 cm^−1^ to 1025.0179 cm^−1^, while another peak shifted from 674.6481 cm^−1^ to 667.193 cm^−1^. MBG-F compositions exhibited similar peak shifts, indicating comparable dissolution behavior to MBG-P compositions. For instance, in MBG-F80, peaks shifted from 1054.8366 to 1028.7452 cm^−1^ and from 674.6481 to 667.1935 cm^−1^. However, MBG-C85 and MBG-C80 showed no shift in the peak observed around 1073 cm^−1^, even after immersion in SBF. In contrast, MBG-C compositions with lower SiO_2_ content, such as MBG-C70 and MBG-C60, demonstrated shifts from 1073.4733 to 1028.7452 cm^−1^and from 674.6481 to 667.1035 cm^−1^. This is explained by the higher surface area of MBG synthesized using Pluronic compared with the other MBG synthesized which means there are more nucleation sites that allow the development of HCA on the surface and faster dissolution.^14,37–39^

Notably, all compositions of MBG exhibit bioactivity after 7 days, as indicated by the emergence of characteristic peaks attributed to P-O bending and stretching vibrations. Comparing the different compositions, it is observed that MBG compositions with 85% SiO_2_ content, which have a higher percentage of bridging oxygens, exhibit lower intensity peaks. The introduction of network modifiers like CaO enhances bioactivity by introducing more non-bridging oxygens to the structure.^14,31^ Turdean-Ionescu et al. discussed in their paper how HCA deposition on the surface of MBG enhances with the increase of Ca and P content in the MBG compositions further highlighting how composition plays an important role in determining the bioactivity of MBG.^37^

**Figure 3.**
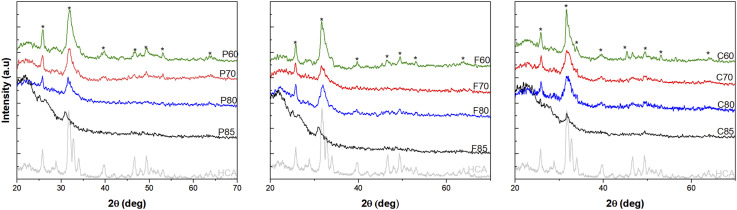
XRD of MBG-F60, MBG-P60, MBG-C60, MBG-F80, MBG-P80 and MBG-C80 before and after immersion in SBF for 7 days. The XRD shows peaks that are representative of the development of HCA on the surface which indicates bioactivity. The peaks that represent the planes of HCA are represented by an *.

XRD analysis presented in Figure 3 illustrates the development of HCA on the surface of MBG after immersion in SBF for 10 days. The distinct peaks observed in the XRD patterns correspond to the characteristic diffraction planes of a hydroxyapatite-like apatite phase, with the asterisks specifically marking the peaks that can be indexed to the reference diffraction pattern for crystalline hydroxyapatite (JCPDS PDF no. 01-086-0740).^13^ The observed peaks at approximately 2θ values of 25.7°, 31.8°, 39.8°, 46.7°, 49.48°, 53.08° and 63.62° degrees for MBG-P60 are characteristic reflections of HCA crystals.^37,40^ These peaks were also observed in other MBG-P compositions as well as MBG-F and MBG-C. HCA commonly exhibits peaks corresponding to the (002), (211), (310), (222), and (213) crystallographic planes^35,36^ which is also shown in the figure. Comparing the different compositions, 60% SiO_2_ MBG shows the most intense peaks that correspond to the HCA pattern, and they show more crystallinity. It is also observed that 70% SiO_2_ have prominent peaks that matches the HCA peaks at 25.75°, 31.84°, 39.64°, 46.68°, 49.42°, 53.18° and 64.02° for MBG-P70 which also match the spectra of HCA observed. It is also observed that MBG-F70 has less notable observed peaks representative of the development of HCA on its surface than the other two compositions with 70% SiO_2_.

80% SiO_2_ MBG had the least crystallinity overall which means that HCA develops slower on their surface. This is in accordance with previous studies such as Dang et al. (2020) where they observed peaks at the same 2θ attributed to the development of HCA on the surface of the MBG.^31^ This was also observed in Turdean-Ionescu where the compositions with higher SiO_2_ showed fewer notable peaks at day 7 than the other compositions and they concluded that MBG that has more Ca and P in their composition produces the largest amount of HCA.^37^

### SEM results

**Figure 4.**
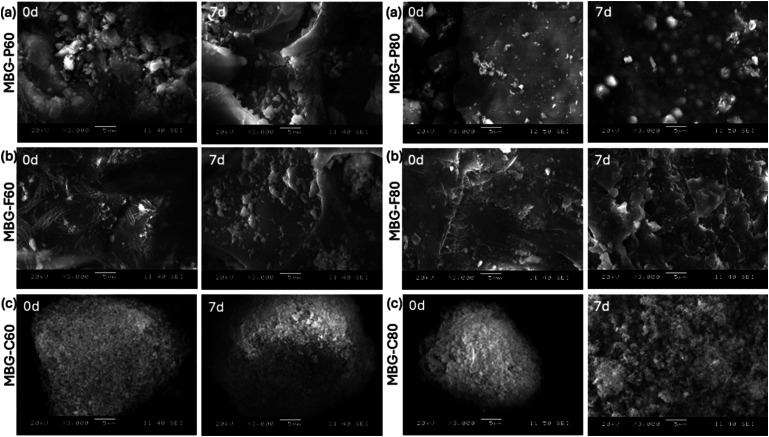
SEM images of MBG that show the glass particle after immersion in SBF for 7 days (a) shows MBG-P60 at day 0 and day 7, (b) shows MBG-F60 at day 0 and day 7, (c) shows MBG-C60 at day 0 and day 7. While (d) shows MBG-P80 at day 0 and day 7, (e) shows MBG-F80 at day 0 and day 7, (f) shows MBG-C80 at day 0 and day 7.

SEM images in Figure 4 demonstrate the progression of HCA formation on the surface of MBG over time when immersed in SBF. Figure 4(a) shows MBG-P80 before and after immersion in SBF for 7 days which shows an irregular and rough structure of the MBG before immersion in SBF with fragments scattered around the bigger particle. However, after immersion in SBF, SEM shows smaller rounder particles that are developing on the surface of the MBG suggesting the biodegradation of the biomaterial and the development of HA on the surface. Figure 4(b) shows MBG-F80 before and after immersion in SBF, which at day 0 shows a porous network and irregular structure but after immersion in SBF, the structure shows increased porosity and erosion of the surface. Finally, Figure 4(c) shows MBG-C80 before and after immersion in SBF for 7 days. The first image was able to capture a single particle around 5 microns that shows the highly porous structure of the MBG-C80, morphologically the particle is more globular and compact compared to the other MBGs. After 7 days, the surface shows the degradation of the biomaterial and the development of HCA on the surface. Similar observations were detected when looking at the SEM of the other compositions where crystalline particles appear on the surface of the MBG after immersion in SBF.^41^

One of the most notable observations from the SEM analysis is that MBG-C often produces uniform, smaller particles compared to MBG-P and MBG-F. This trend is also evident in the particle size distributions. While MBG-P and MBG-F frequently exhibit rougher and more irregularly shaped particles, MBG-C particles tend to be more uniform and smaller. This difference can be attributed to the micellization behavior and surface charge of the surfactants used. CTAB, a cationic surfactant, forms smaller and more uniform pores than Pluronic surfactants, which are non-ionic and typically generate larger micelles.^18^ During the synthesis of MBG-C, ammonia serves as a catalyst, facilitating the formation of spherical micelles by CTAB. In this process, the hydrophilic head of CTAB faces the solution, while the hydrophobic tail points inward. Consequently, as the MBG forms around the micelle, the metal oxide precursors interact directly with the polar head, leading to the formation of silica around the micelle structure.^13^ While in the case of Pluronic surfactants, the synthesis occurs in more acidic solutions where the formation of silica around the micelle is slower and more stable. This slower. Reaction kinetics lead to the development of more irregular shapes in the resultant MBG. Pluronic surfactants can micellize in wide pH range including more acidic environments which causes a slower and more controlled formation of silica which can result in irregular shapes.^12^

The steady dissolution and biodegradation seen *in vitro* indicate promising bioactivity that should be further examined *in vivo* to assess interaction with physiological fluids and remodelling capacity.^31,39^ This favourable degradation profile supports the release of loaded antibiotics from the MBG matrix during dissolution, enabling local delivery in defects to potentially treat challenging infections.^41,42^

### Induced coupled plasma analysis

**Figure 5.**
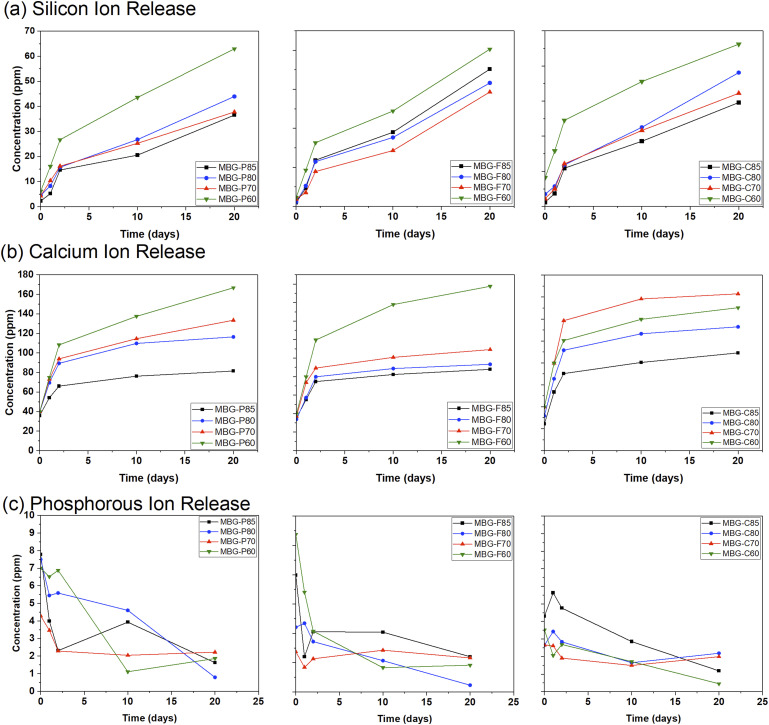
Si, Ca, and P ion release after immersion of MBGs in SBF for different periods. (a) The release of silicon ions from the different compositions is compared to each other to show any clear trends that indicate biodegradation of the MBG. (b) Release of calcium ions, which is indicative of bioactivity and the formation of HA, and (c) release of phosphate, which, similar to calcium, shows the bioactivity of the synthesized bioglass with an error average < 5%.

The data collected in Figure 5 show the ion release from MBG-P, MBG-F, and MBG-C and their different compositions. Figure 5(a) specifically looks at the silicon release from the different compositions; all MBG tested show an overall trend of increasing Si release over time as the glass degrades in SBF. Starting with MBG-P, it observed that MBG-P60 has the highest levels of Si released overtime, followed closely by MBG-P80 and MBGP-70 which both showing similar release of Si ions and trailing by MBG-P85. For MBG-F MBG-F60 has the highest release followed closely by all the other compositions. Finally, for MBG-Cs, MBG-C80 shows the highest releasee followed by the other compositions showing almost identical trends of Si release overtime. This trend is also observed for the other MBGs which makes sense as 60% SiO_2_ will release Si ions more easily as the addition of CaO to the structure of the glass leads to more non-bridging oxygens in the structure and causing more rapid release of ions than MBG with a higher SiO_2_ and therefore more bridging oxygens.^14^

Figure 5(b) shows the release of calcium ions overtime from the different MBGs tested. It is observed that all different MBG compositions show an initial burst release of Ca followed by a plateau at day 2 which continues to the end of the testing on day 20. The general trends show that the impact of composition is the determining factor in the release of Ca ions where 60% SiO_2_ tend to release the most Ca ions followed by 70% SiO_2,_ > 80% SiO_2_ > 85% SiO_2_. This makes sense as compositions with lower silicon oxide molar ratio, have a higher percent of calcium in their structure.^43^

Figure 5 (c) looks at the release of phosphorus ions from the different MBG compositions. In general, P ions release decreases overtime where at its highest it starts at 8-9 ppm and ends at ∼1 ppm by the end of the testing period. These results agree with previous studies on MBG which also indicate that phosphorous concentration decreases overtime.^16,44,45^ When looking at different compositions, it is observed that no common theme can be established among the different MBGs; for MBG-P the highest drop in P ion concentration can be observed in MBG-P60 while MBG-P85 initially decreases then increases slightly only to decrease again after day 10. MBG-Fs show very similar P ion release among all compositions where there is a small difference between the initial and final concentrations overall. Meanwhile, MBG-Cs show the highest drop in P ion concentration initially compared to final concentration. It is important to note that all different compositions had the same initial P_2_O_5_ in the compositions and that differences shown here are expected due to the stability of the MBG network when introducing non-bridging oxygens and choice of surfactant. In conclusion, P ions overall have lower release with higher SiO_2_ molar ratio in the composition, which leads to higher stability overall of the structure. Comparing the different surfactants shows that MBG-F have similar P ion release among all compositions, while MBG-P has the most variability between initial and final concentration, compared to MBG-C which shows a sharp decrease in P concentration across all compositions.

Recent studies, such as the work by Schumacher et al., highlight the challenges of directly comparing ion release data across different experimental setups and materials compositions.^14^ However, some general trends can be observed that agree with the literature. Prior studies show that higher calcium and phosphorus release from MBG compositions with lower SiO_2_ content which agrees with the results observed in this study.^17^ The effect of surfactants during synthesis on subsequent ion release kinetics remains relatively unexplored. The results of this study suggest that surfactants do not have a strong impact on ion release while compositions were the main factor impacting ion release across all synthesized MBG.

### Surface area analysis

**Figure 6.**
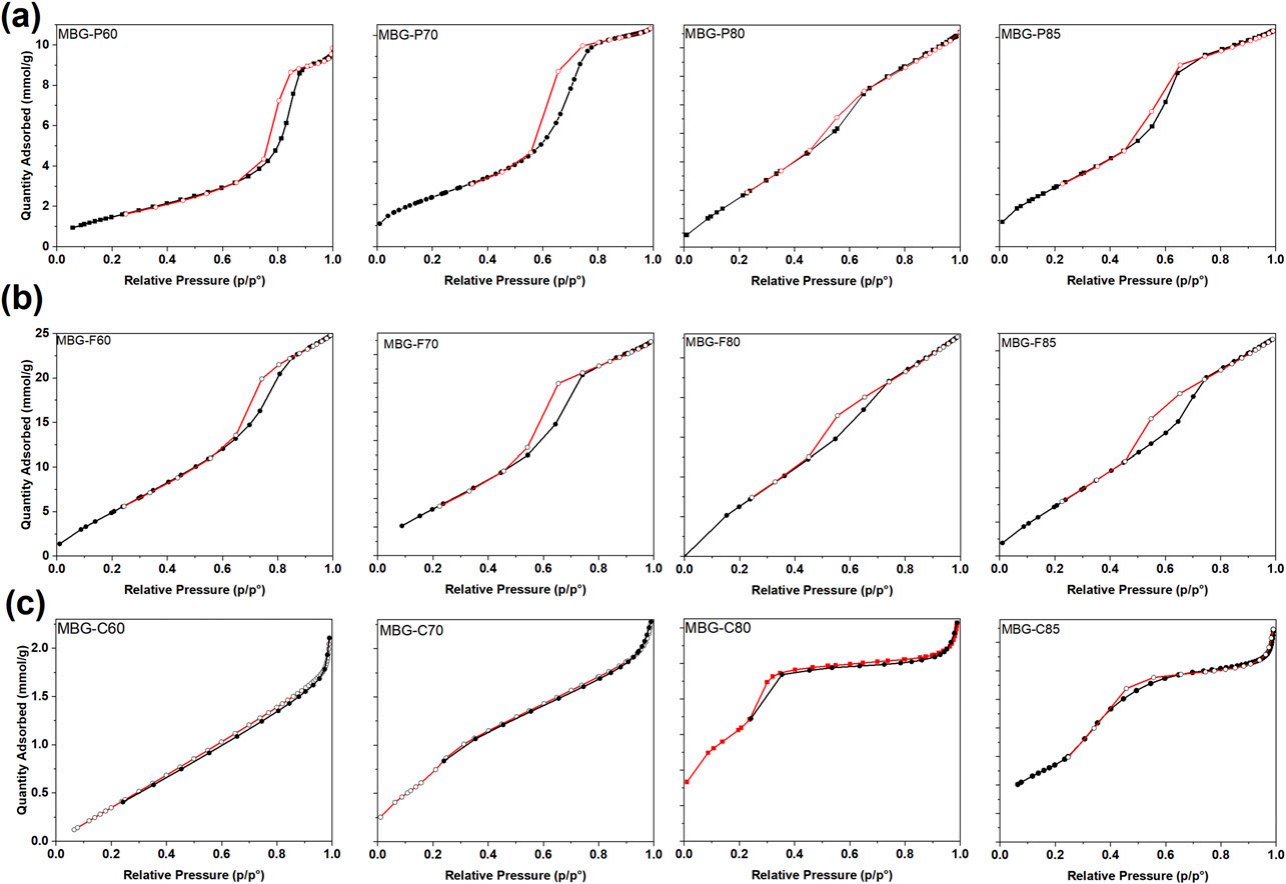
Nitrogen adsorption-desorption isotherm showing the quantity of gas adsorbed (cm^3^/g) as a function of pressure (p/p^o^). (a) is the N_2_ adsorption isotherm for MBG-P. (b) is the N_2_ adsorption isotherm for MBG-F. (c) is the N_2_ adsorption isotherm for MBG-C.

The nitrogen adsorption-desorption isotherms in Figure 6 provide insights into the mesoporous structure and textural properties of the synthesized MBGs. All MBG-P compositions exhibit a type IV isotherm, which is characteristic of mesoporous materials. MBG-P70 shows a clear H_1_ hysteresis loop, indicative of uniform cylindrical pore channels, forming between relative pressures of 0.6 and 0.8 (p/p°). Similarly, MBG-P85, Figure 6(b), also exhibits a type IV isotherm with an H_1_ hysteresis loop observed between relative pressures of 0.5 and 0.7 (p/p°), suggesting a similar pore structure but with potential differences in pore size or volume. These hysteresis loops confirm the mesoporous nature of the materials, with MBG-P compositions exhibiting consistent pore characteristics across varying surfactant concentrations. The observed shifts in hysteresis behavior, particularly for MBG-P60, highlight the impact of synthesis conditions on pore formation, as evidenced by H_2b_ hysteresis observed at higher relative pressures 0.7–0.9 (p/p°) for MBG-P60 which forms an ink-bottle shaped pores.^46^

Figure 6(b) shows the nitrogen adsorption desorption isotherms for MBG-F which like MBG-P, all exhibit type IV isotherm indicative of mesoporous material. MBG-F70, MBG-F80 and MBG-F85 all show a H_1_ hysteresis loop as evidenced by the loop created between relative pressure of 0.5-0.7 (p/p°). This type of hysteresis is formed when uniform cylindrical mesopores channels are created.^14^ However, for MBG-F60 the hysteresis formed around 0.7–0.9 (p/p°) which resembles the hysteresis created by MBG-P60 meaning that the impact of introducing network modifiers, by increasing calcium oxide molar percentage, in the structure also impacts the pores formed by the MBG.

Figure 6(c) shows the nitrogen adsorption desorption of MBG-C which is noticeably different from the MBG synthesized using the Pluronic surfactant. As discussed before, CTAB forms uniform spherical micelles in the solution and creates relatively smaller MBG particles than the other surfactants. MBG-C still shows a type IV isotherm with H_2_ hysteresis but with smaller hysteresis that is probably caused by the overall smaller structure of the CTAB which means that it produces narrower mesopores and non-uniform pores.^47^

The analysis shows that all MBG types formed a type IV isotherm confirming mesoporosity via multilayer adsorption followed by capillary condensation, the hysteresis is formed when there is capillary condensation which limits the gas uptake.^15^ Similarly, most compositions in this work showed characteristic type IV profiles featuring H1 hysteresis loops, indicative of uniform cylindrical mesopore channels from 2 to 50 nm.^14,15^ The presence of hysteresis signifies capillary condensation inside the mesopores at high relative pressure and subsequent evaporation of adsorbed nitrogen upon desorption.^15,46^ Sharp desorption isotherms signify highly uniform pore sizes and cage-like pores. Variations were observed between surfactant systems, with MBG-Ps and MBG-Fs showing H_1_ loops corresponding to cylindrical channels, while the MBG-C compositions exhibited H_2_ hysteresis, suggesting more interconnected mesopores shaped by wormholes.^48^

Table 2 looks at the porosity of the MBGs including pore volume, pore sizes and surface area of the different MBG synthesized. The porosity of the materials is often classified based on their ability to adsorb gases on the surface. The amount of gas adsorbed to the surface of the porous materials at a fixed temperature as a function of the pressure.^15^ Upon evaluating compositions used in this study, it’s evident that MBG-P80 has the highest surface area, coupled with the largest pore size and volume. Subsequently, the MBG-F80 and MBG-C80 have very similar surface area but MBG-F80 has larger pore size and volume. The comparison between MBG-P70 and MBG-C70 showcases a similar trend, with MBG-P70 exhibiting a greater surface area and pore volume, while MBG-C70 presents a larger pore size. This trend persists across MBG-P60 and MBG-F60, revealing that MBG-P again has a higher surface area, yet MBG-F60 showcases a higher pore volume and size. Across different compositions, the Pluronic P123 series—from MBG-P60 to MBG-P85 illustrates a clear hierarchy in surface area, with values of 695.2 m^2^/g, 651.1 m^2^/g, 647.7 m^2^/g, and 439.2 m^2^/g respectively. A higher surface area predicts an increased formation of HCA, which enhances bioactivity.^47^ This is supported by the data presented in Figures 2 and 3 where MBG-P consistently shows the highest bioactivity among different MBGs.

**Table 2.** Pore size, pore volume, and surface area of different compositions of MBG.

Composition	Pore size (nm)	Pore volume (cm^3^/g)	BET surface area (m^2^/g)	Particle distribution (m)	Drug loading (%)
MBG-P85	5.73	0.71	439.2	2.287 ± 0.44	33.19 ± 5.34
MBG-P80	5.17	1.28	695.3	2.969 ± 0.82	74.58 ± 2.71
MBG-P70	3.80	0.89	651.1	2.901 ± 0.63	83.75 ± 2.46
MBG-P60	2.77	0.81	647.7	1.416 ± 0.01	10.45 ± 2.15
MBG-F85	5.51	1.09	561.8	2.467 ± 0.17	27.58 ± 3.99
MBG-F80	4.73	0.95	490.2	1.658 ± 0.16	77.06 ± 1.56
MBG-F70	6.27	0.51	252.6	5.423 ± 0.2.36	47.72 ± 0.58
MBG-F60	6.51	0.85	381.4	9.812 ± 0.1.84	58.22 ± 5.06
MBG-C85	4.42	0.25	223.73	4.856 ± 0.57	21.29 ± 0.21
MBG-C80	3.10	0.41	489.5	6.616 ± 0.48	63.11 ± 3.77
MBG-C70	4.35	0.76	575.6	11.42 ± 1.14	10.00 ± 2.75
MBG-C60	4.18	0.07	64.12	6.856 ± 1.66	10.90 ± 4.87

Within MBG-F, MBG-F85 exhibits the highest values in surface area, pore size, and pore volume, followed by MBG-F80, MBG-F60, and then MBG-F70. In the MBG-C range, MBG-C70 surpasses MBG-C80 in surface area, pore size, and volume, while MBG-C60 records the lowest surface area in all compositions. Overall, these synthesized glass compositions align closely with values previously documented in the literature. Wu et al. (2011) have listed the surface area, pore volume, and pore size of a few MBG compositions in their review with numbers that correspond to the numbers observed in this study. Pluronic P123 and F127 produced MBG with a surface area of 300–520 m^2^/g^10^ which are similar to the values reported here. Yan et al. (2006) and Zhao et al. (2008) reported similar pore sizes as observed here with a range of 4.9–6.1 nm for MBG-P but reported a lower surface area range of 146–351 m^2^/g.^12,49^ Anand et al. found the surface area of MBG synthesised with CTAB and a composition containing 78% SiO_2_ content to be around 470 m^2^/g which is very close to MBG-C80 reported here.^43^

Particle size distribution of the different MBG was also measured to compare the different compositions and surfactants used. For MBG-P, the particle distribution ranged from 1.41 to 2.9 micron while the particle distribution for MBG-F and MBG-C ranged from 1.65 to 9.81 and 4.8-11.42 respectively. This indicates that all particles observed through DLS were between 1 and 11 microns and can be related to the surface area observed for each.

### In vitro drug release

#### Drug loading

Table 2 also shows that both the composition MBG and the surfactant template have a significant influence on vancomycin loading capacity. MBG-Ps generally showed higher loading across compositions, with the highest being MBG-P70 which achieved a loading of 83.75% followed by MBG-P80 (74.58%). Among the MBG-P series, the lowest loading was achieved in MBG-P60, which had 10.48% drug loading efficiency. MBG-F had more variability, with highest loading for MBG-F80 (77.06%) but lowest for MBG-F85 (27.58%) while MBG-F70 and -F60 achieved similar loading efficiency of 47.72% and 58.22% respectively. The MBG-Cs also exhibited composition dependence but had a markedly lower overall load, below 63% for all versions and low for MBG-C70 (10%). Furthermore, a composition dependence effect is observed, as the 60% SiO_2_ MBG consistently had lower drug loading (10%–58%) compared to the 70%–85% SiO_2_ compositions in each surfactant systems.

These drug delivery performance differences can be attributed to the combined impact of textural properties and chemistry changes from both the inorganic framework and surfactant templates during synthesis as well as surface area. During synthesis the micellization of surfactants depends on their specific properties which in turn influences the final structure of the MBG. After the successful synthesis of MBG and complete template removal, the MBG exhibits different characteristics based on the template used, including variations in mesopores sizes and volumes^10^ which undoubtably impacts drug loading and release. Overall, the results in Table 2 demonstrate that the adjustment of the composition of MBG and the selection of surfactants enables the optimization of the drug loading efficiency. The value of 60% SiO_2_ MBGs exhibit a lower surface area in the range of 381.4–648 m^2^/g, with pore sizes between 2.8 and 6.5 nm except for the CTAB-based C60 that has a markedly lower surface area of 64 m^2^/g and a pore volume of 0.07 cm^3^/g pore volume. This correlates with a poor drug loading of 10%–58%. In comparison, 70%–85% of SiO_2_ MBGs achieve higher surface areas of 223–695 m^2^/g and pore volumes between 0.25 and 1.28 cm^3^/g leading to excellent 21%–83% vancomycin loading. The very high loading and surface area achieved with 80% SiO_2_ across the board signifies an optimal balance of surface area and stability is reached at that composition. Overall, the results show that the tuning of MBG texture and chemistry has a coupled effect that enables substantial control over the ensuing drug delivery capability.

**Figure 7.**
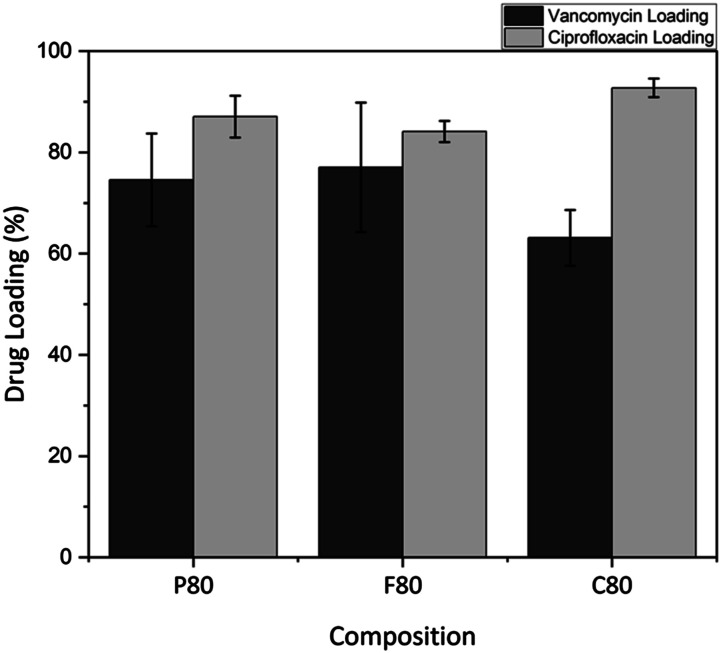
Drug loading (%) of vancomycin (orange) and ciprofloxacin (green) on MBG-P80, MBG-C80 and MBG-F80. Ciprofloxacin had a higher loading rate for all compositions.

Ciprofloxacin is a broad-spectrum fluoroquinolone antibiotic often used to treat various infections affecting the joints, bones, and other sites.^50^ Comparing the drug loading capacity of ciprofloxacin to vancomycin shows a strong dependence on the specific drug used. Ciprofloxacin exhibited higher loading across all the compositions tested, with an average loading of 80%–96% versus 63%–77% for vancomycin as shown in Figure 7, this was done by immersing three MBG samples into each drug solution and measuring the loading using UV-VIS. The bar graph also compares the drug loading efficiencies of vancomycin and ciprofloxacin across the different MBG samples tested. An analysis of the standard deviation indicate that vancomycin loading had more variability than ciprofloxacin loading. For example, MBG-P80 vancomycin reached 87.2% in one trial but dropped as low as 69.43% and 74.58% in others, reflecting notable fluctuation in loading efficiency. In contrast, ciprofloxacin loading across all MBG types had smaller error bars, indicating less variability and more consistent loading. This difference is explained by the smaller particle size of ciprofloxacin compared to vancomycin.^51^ The smaller ciprofloxacin particles can more easily penetrate the mesopores and adsorb onto the large surface area of the MBG, leading to a greatly increased loading over the larger vancomycin molecules. The results demonstrate that in addition to MBG composition and surfactant selection, the physicochemical properties of the drug itself, such as particle size, significantly influence the achievable drug loading.^51^

The drug-loading ability of mesoporous materials depends on the drug itself, and the properties of the materials being tested. Vancomycin which was used in this study is often used to treat infections caused by harder-to-treat bacterial infections. It is a glycopeptide antibiotic that is most effective against gram-positive bacteria such as *Staphylococcus aureus* and is often used to treat bone infections.^25^ Vancomycin has a relatively large particle size compared to other antibiotics which means that a smaller pore size will not allow for high loading of the antibiotic compared to trying to use an antibiotic with a smaller particle size. It is assumed that most of the vancomycin loaded into the MBG was adsorbed to the surface and did not form hydrogen bonds with the MBG. The surface charge of MBG is a critical factor that influences drug loading and release. According to Jiang et al. and Lee et al., MBG typically exhibits a negative surface charge, which is also presented in many biological molecules and drugs.^52,53^ This negative surface charge can impact the efficiency of drug loading onto the MBG. To optimize drug loading, functionalization of the MBG surface could lead to higher drug loading. Functionalization of the surface aims to create stronger bonds between the drug and the MBG. This approach is expected to lead to prolonged drug release, enhancing the therapeutic efficacy of the loaded drugs.

#### Drug release

**Figure 8.**
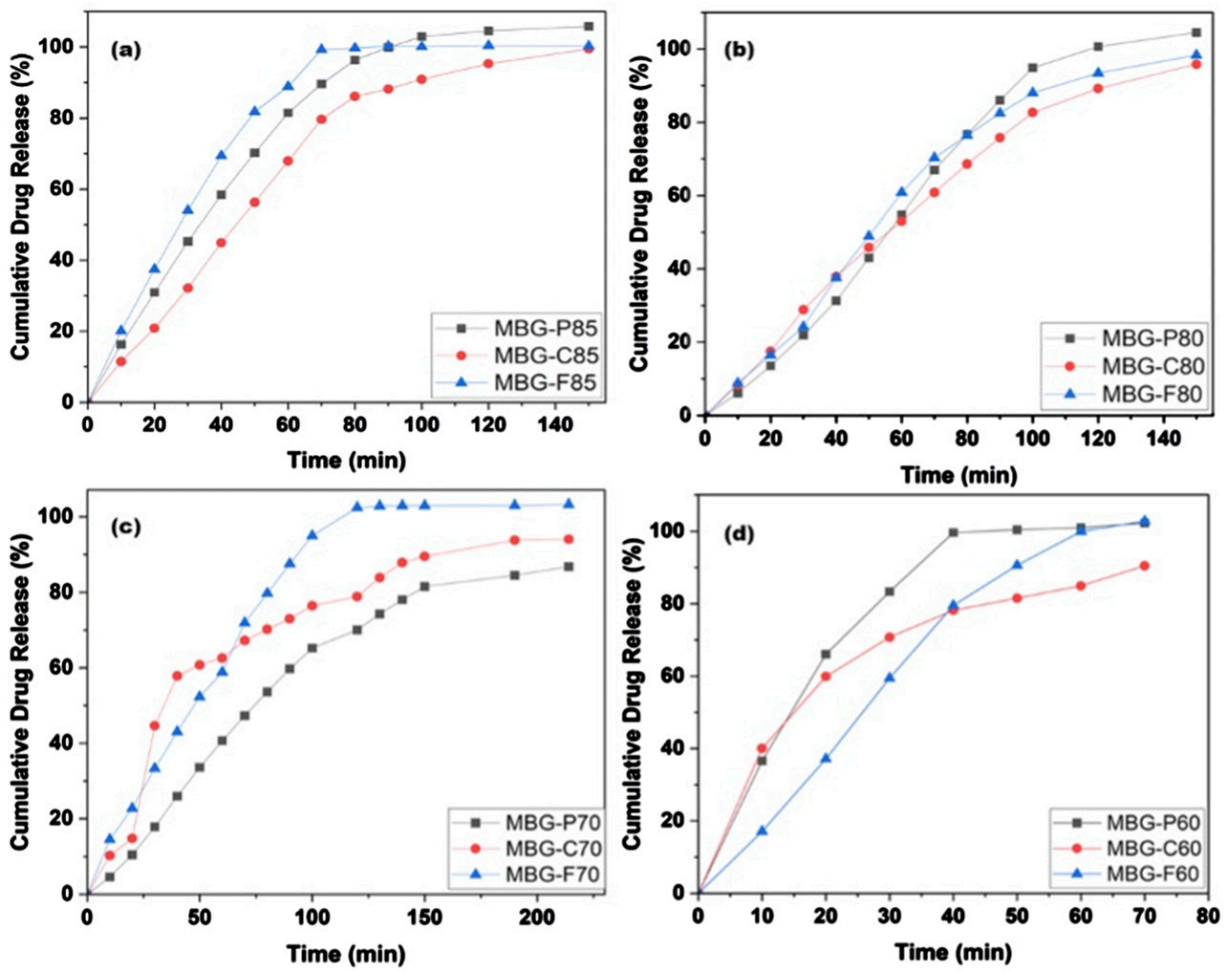
Cumulative vancomycin release from (a) MBG-P85, -F85, and -C85, (b) MBG-P80, -F80, and -C80, (c) MBG-P70, -F70, and -C70, and (d) MBG-P60, -F60, and -C60 with error average of 6%.

Figure 8 shows the *in vitro* release kinetics of vancomycin from the MBG compositions showed some differences depending on the surfactant template used during sol-gel synthesis. Figure 8(a) shows that MBG-F85 has the fastest release among all the 85% silica MBG as it was able to release all the loaded vancomycin within 80 minutes of the start of the experiment. MBG-P85 and MBG-C85 show similar release with both reaching 100% release within 140 minutes slightly slower than the MBG-F85 counterpart. Figure 8(b) shows the release of vancomycin from MBG with 80% silica, which shows that all different MBGs have very similar release rates with all of them reaching around 100% release by 150 minutes. This indicates that the release is more dependent on the loading rate rather than the composition or surfactant used. For Figure 8(c) MBG-F70 showed the quickest release reaching 100% around 120 minutes while MBG-P70 and MBG-C70 reaching 70% and 78% by that time. Figure 8(d), displayed the most rapid release for all compositions, eluting most loaded vancomycin within 70 minutes, faster than all other MBGs. MBG-P60 reached 100% by minute 50 while MBG-C60 and MBG-F60 reached 100% by 70 minutes.

In comparison across the MBG compositions, the lower silica content MBGs (70%–80% SiO_2_) enabled more gradual, sustained vancomycin release over 120+ minutes rather than rapid burst release. The highest 85% SiO_2_ formulations displayed the fastest complete elution within 80-150 minutes. While by observing surfactant effects, F127 promoted the highest drug delivery across varying compositions while P123 and CTAB generated lower extended releases for a given SiO_2_ percentage. Therefore, both MBG composition and surfactant template played an important role in modulating the release kinetics. For most compositions, the release ranked: F127 > P123 > CTAB, revealing intrinsic effects based on surfactant properties. This agrees with the BET results showing the F127-templated MBGs achieve larger pore sizes than the other surfactants. In comparison, P123 has lower hydrophilicity and a smaller micelle structure, resulting in smaller pores and supporting more sustained diffusion properties.

Specifically, when looking at the release rate in Figure 8, it is noted that there are several factors impacting the release of drug from different MBG. While the surfactant type plays a role in shaping the porous structure and influencing drug loading, the drug release profiles are more closely tied to a combination of drug loading capacity and the MBG composition. Notably, MBGs with the lowest silica content demonstrated the fastest drug release rates among all samples, suggesting that the matrix degradation plays a role in lower silica formulations. Conversely, the MBG with the highest silica content exhibited the second-fastest drug release rate, likely due to its relatively low drug-loading capacity, which limits sustained release. The figure also shows the MBG with 80% silica had the most consistent release where the three types showed very similar release reaching around 100% within 150 minutes. In contrast, MBGs with 70% silica content showed significant variability in release rates, reflecting differences in initial drug loading. For example, MBG-P70 achieved an exceptionally high drug-loading efficiency of 83.75%, while MBG-C70 exhibited a low loading efficiency of just 10%. This disparity is mirrored in the release profiles, with MBG-P70 showing a more sustained release due to its higher drug reservoir, while MBG-C70 released its drug content more rapidly. These results emphasize the importance of balancing MBG composition, drug loading, and release kinetics for optimizing drug delivery systems.

The relationship between MBG composition, surfactant type and drug release are multifactorial, with both factors contributing to release kinetics. Lower silica content in 70%–80% SiO_2_ MBG demonstrated more gradual and sustained drug release over 120 minutes whereas higher silica content (85% SiO_2_) exhibited faster elution within 80–150 minutes, due to differences in drug loading ability and matrix degradation. When looking at surfactants, F127 consistently promoted the highest drug release across all compositions, attributed to its larger pore sizes (average pore size of 5.76 nm), while P123 and CTAB with smaller pores (average pore size of 4.36 nm and 4.01 nm respectively) had a slower release. This means that drug loading and release is mediated by surfactant-mediated pore structure and silica content.

**Figure 9.**
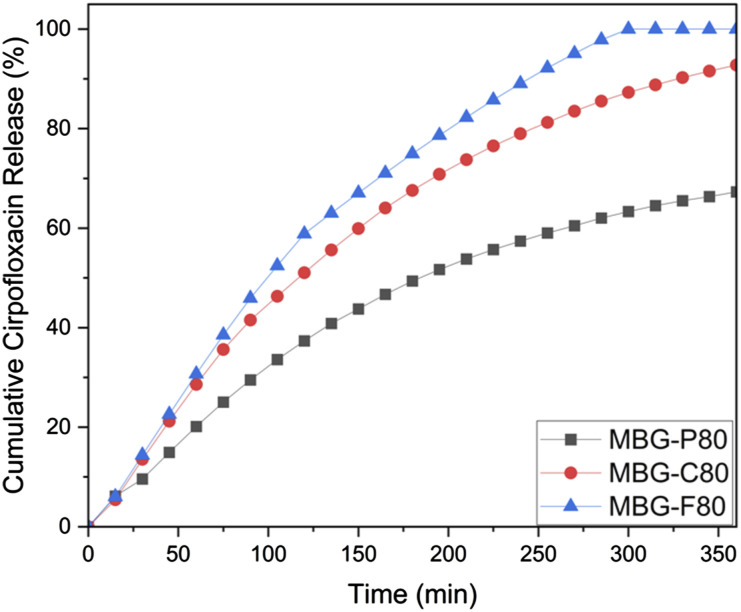
Cumulative ciprofloxacin release from MBG-P80 (black), MBG-C80 (red) and MBG-F80 (blue) with error average of 5%.

Figure 9 illustrates the ciprofloxacin release profiles for MBG-P80, MBG-C80, and MBG-F80. All MBGs exhibit a burst release, with MBG-F80 displaying the most rapid release within the first 6 hours. In contrast, MBG-C80 and MBG-P80 demonstrate a slower, more sustained release. This is consistent with previous findings that ciprofloxacin had relatively higher loading efficiency in all the tested MBGs, which explains the more prolonged release behaviour. Within the first 6 hours, only MBG-F80 achieves 100% release, whereas MBG-C80 reaches 92%, and MBG-P80 reaches about 70%. This is different from the release data for vancomycin, where all MBGs achieve complete release within 3 hours. The faster release rate of vancomycin across all compositions suggests that it remains on the MBG surface, whereas ciprofloxacin is encapsulated within the MBG pores, resulting in a more gradual release. The average error rate was calculated for MBG-P80, MBG-C80, and MBG-F80, with each demonstrating an average error rate of 6.4%, 7.5%, and 5.4%, respectively. These variations in error rates suggest a high level of reliability in the release measurements, with MBG-P80 showing the lowest variability, followed by MBG-F80 and MBG-C80. This further supports the consistency of the observed release profiles and highlights the reproducibility of the experimental results.

Table 3 shows the correlation coefficients for various drug release kinetics to assess the release from the different compositions. The fit was evaluated through the coefficient of determination, R^2^ which is value that determines the goodness of fit of drug release kinetic models. A higher R^2^ value indicates that the model fits the experimental data better which means that the best fit will have a higher R^2^ value.^27^ After fitting the different release profiles to drug release models including zero-order, first-order, Higuchi, and Korsmeyer-Peppas models^27,28^ it was observed that most of these MBGs had a high coefficient value that matches the zero-order model, indicating that drug adsorbed are released at a constant rate irrespective of the remaining concentration, which is expected for highly porous materials. However, this mostly deviates for the 60% SiO_2_ formulations, with R^2^ values dropping to 0.858 and 0.8194 for MBG-P60 and MBG-C60. The Higuchi model provides the best fit for these compositions, indicating diffusion-based release from low-silica MBG. However, MBG-F60 maintains predominant zero order release. Izquierdo-Barba et al. (2015) reviewed how the porous structure, composition, and surfactant templating of MBGs can impact drug release kinetics.^34^ This is highlighted in the present study, where fitting the release data to different mathematical models confirms that MBGs do not predominantly follow a single release mechanism. Rather, the release kinetics are governed by multiple coupled factors related to MBG synthesis, which provides opportunities for tailored optimization. By altering composition or surfactant selection, the drug release profiles can be tuned to exhibit different rates and dynamics depending on the intended application.

**Table 3.** Comparison of drug release kinetics using various models and corresponding correlation coefficients for model fit assessment.

Sample name	Zero-order	First-order	Higuchi	Korsmeyer-Peppas
	R^2^	R^2^	R^2^	R^2^
MBG-P85	0.996	0.9694	0.9409	0.8184
MBG-C85	0.9988	0.9666	0.8999	0.7565
MBG-F85	0.9835	0.995	0.9563	0.8479
MBG-P80	0.9865	0.9499	0.8443	0.6833
MBG-C80	0.9964	0.9944	0.9175	0.7826
MBG-F80	0.9896	0.9513	0.8613	0.706
MBG-P70	0.9937	0.9807	0.8646	0.7074
MBG-C70	0.9188	0.9318	0.8631	0.7427
MBG-F70	0.9918	0.9859	0.9506	0.8354
MBG-P60	0.858	0.8001	0.9684	0.946
MBG-C60	0.8194	0.966	0.9774	0.9899
MBG-F60	0.9838	0.6793	0.9313	0.8094

Examining the influence of surfactants, it appears they had minimal effect on the release kinetics of vancomycin from MBG, with zero-order release observed in all but four compositions. These four compositions exhibited lower loading rates compared to others, potentially explaining their reduced release rates. To further explore the surfactant’s impact on drug loading and release, the release rate of ciprofloxacin-loaded samples was also analyzed. After fitting the data to various models, it was noted that MBG-P80 demonstrated the best fit with first-order release, although this was not significantly *better* than fits of the other models. MBG-F80 and MBG-C80 showed first-order and zero-order kinetics, respectively. This, along with the observation that ciprofloxacin generally had higher loading across all compositions compared to vancomycin, suggests that the drug itself also influences release kinetics. The first-order release mechanism from bioactive glasses often involves drug molecules diffusing out of the glass’s porous structure into the surrounding medium, possibly due to weaker ionic interactions between ciprofloxacin and MBG. Understanding the interplay between the drug and MBG can aid in designing functionalized MBGs with strong drug bonds, enabling slow release for treating challenging infections.

**Figure 10.**
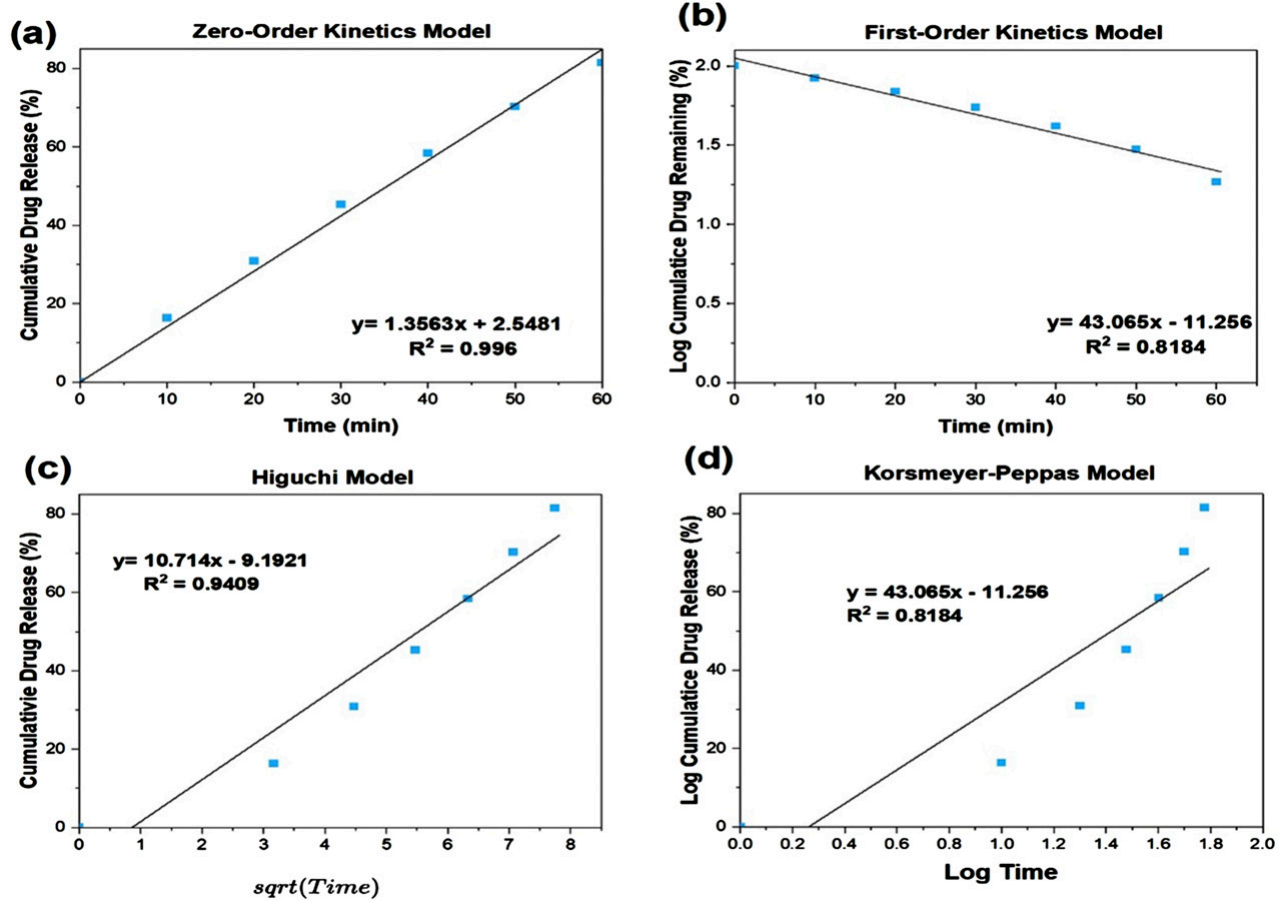
Fitting of vancomycin release data from MBG-P85 to various mathematical models. The coefficient of determination (R^2^) was determined for each model to determine the goodness of fit. Vancomycin release from all MBG compositions was fitted to (a) zero order, (b) first order, (c) Higuchi, and (d) Korsmeyer-Peppas models similar to this example fitting.

Figure 10 shows an example of how the data was fitted through the different kinetic models. Each sample was fitted, and the correlation coefficient was obtained to compare which model was the best fit to represent the release kinetics. Comparison of the R^2^ values enabled the identification of the most suitable model for each MBG formulation that best describes the release kinetics. This exemplary fit of MBG-P85 data demonstrates the process applied for kinetic evaluation and mathematical model selection across all MBG samples.

### Antimicrobial susceptibility test

**Figure 11.**
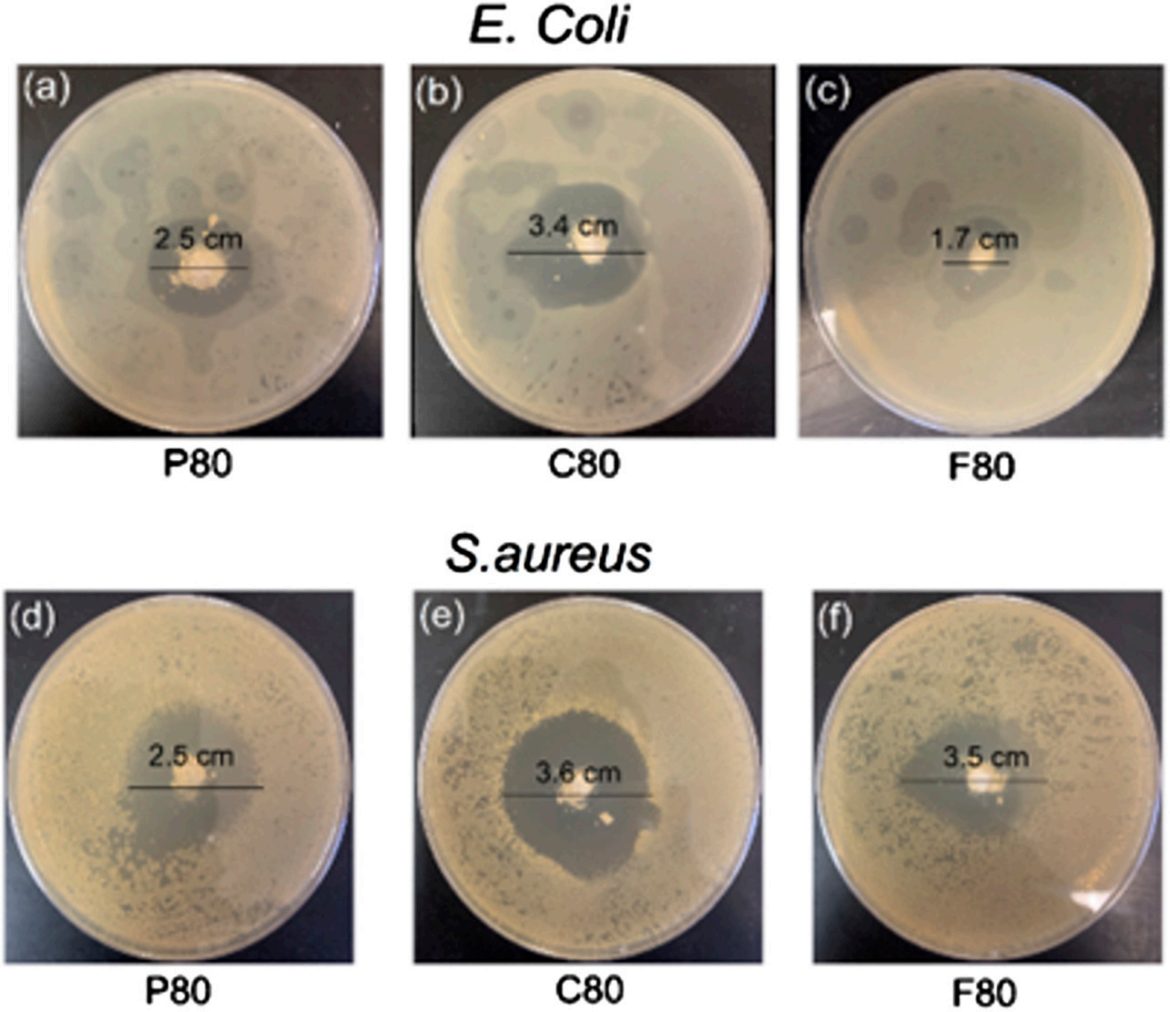
Images of antibacterial testing for vancomycin-loaded MBG against gram-positive *E. coli* and gram-negative *S. aureus*. The images show the zone of inhibition as observed after 24 hours of incubation at 37°C (all measurements ±0.2 cm).

Figure 11 shows the antibacterial efficacy of vancomycin-loaded MBGs evaluated using the agar disc diffusion method against *S. aureus* (Gram-positive) and *E. coli* (Gram-negative). The MBGs were loaded into wells on agar plates, and the zones of inhibition were measured after 24 hours of incubation at 37°C. The results show that MBG-P80 exhibited equal zones of inhibition for both bacteria, indicating similar efficacy. MBG-C80 also displayed comparable antibacterial activity against both strains, suggesting a consistent release of vancomycin. However, MBG-F80 demonstrated a significantly smaller zone of inhibition for *E. coli* compared to *S. aureus*, indicating reduced efficacy against Gram-negative bacteria. These findings suggest that vancomycin-loaded MBGs are broadly effective against both bacterial types, but the efficacy varies with MBG type. The reduced activity of MBG-F80 against *E. coli* may be attributed to differences in drug release kinetics. For MBG-F80, vancomycin remains predominantly on the surface of the MBG particles, resulting in slower or incomplete release into the agar medium. In Figure 8, the cumulative release rate of vancomycin was very similar for MBG-P80, MBG-C80 and MBG-F80 which is why they were chosen for the antimicrobial studies. It can be assumed that any differences observed here are due to MBG properties including inherent antimicrobial properties that are caused by ion release and pH changes.

## Summary and Conclusions

This study looked at the impact of surfactant type and MBG composition on drug loading, release kinetics and bioactivity. The findings indicate that composition plays a more important role in bioactivity; it was shown that MBGs with lower silica content showed higher bioactivity, as demonstrated by SEM, FTIR, and XRD analyses. For drug loading and release, MBGs with 70%–80% silica content had the highest loading efficiency and more sustained release profiles compared to other MBGs. Surfactant Pluronic F127 created larger pore sizes, which led to higher release across all compositions, while surfactants Pluronic P123 and CTAB created smaller pore sizes and different pore structures, leading to slower, more sustained release. The release kinetics showed that most MBGs with high silica content had a zero-order release profile, characterized by a constant drug release rate that is independent of the initial drug loading. In contrast, MBGs with lower silica content predominantly followed a diffusion-controlled release mechanism driven by the degradation of the matrix. Nearly all loaded vancomycin was released within the first 4 hours. Compared to ciprofloxacin, which was slower and more sustained, lasting 6 hours and releasing around 100%, 92% and 70% for each of MBG-F80, MBG-C80 and MBG-P80.

Surfactant type influenced pore size, structure, and particle distribution, as evidenced by BET and nitrogen adsorption analysis. MBG-P and MBG-F, synthesized using Pluronic surfactants, displayed type IV isotherms with H_1_ hysteresis, characteristic of mesoporous materials, whereas MBG-C, synthesized with CTAB, exhibited H_2_ hysteresis, indicating smaller pore sizes. SEM images further revealed that MBGs synthesized with Pluronic surfactants had rough and irregular structures, while MBG-C showed more circular and uniform structures. Bacterial studies demonstrated that all MBG samples were effective against both *S. aureus* and *E. coli*, with similar zones of inhibition observed in disc diffusion tests.

Overall, this study highlights the ability to tailor MBG texture and chemistry through surfactant choice and composition to optimize drug loading, release behavior, and antibacterial performance. In the future, the best-performing MBG compositions and surfactant combinations will be further tested for prolonged drug release and their effects on *in vivo* models to confirm their potential for drug delivery and bone regeneration applications.
